# Every-Other-Day Feeding Decreases Glycolytic and Mitochondrial Energy-Producing Potentials in the Brain and Liver of Young Mice

**DOI:** 10.3389/fphys.2019.01432

**Published:** 2019-11-22

**Authors:** Oksana M. Sorochynska, Maria M. Bayliak, Dmytro V. Gospodaryov, Yulia V. Vasylyk, Oksana V. Kuzniak, Tetiana M. Pankiv, Olga Garaschuk, Kenneth B. Storey, Volodymyr I. Lushchak

**Affiliations:** ^1^Department of Biochemistry and Biotechnology, Vasyl Stefanyk Precarpathian National University, Ivano-Frankivsk, Ukraine; ^2^Department of Neurophysiology, Institute of Physiology, Eberhard Karls University of Tübingen, Tübingen, Germany; ^3^Department of Biology, Carleton University, Ottawa, ON, Canada

**Keywords:** intermittent fasting, glycolytic enzymes, electron transport chain, mitochondrial respiration, ketone bodies

## Abstract

Intermittent fasting is used to reduce body mass in obese adult humans and animals. However, information on the impact of one type of intermittent fasting (IF) called every-other-day feeding (EODF) on young animals is scarce. In this study, 1-month-old mice of both sexes were subjected to a 4-week regimen of EODF using age-matched counterparts fed *ad libitum* as controls. At the end of EODF exposure, experimental male and female mice weighed 14 and 13% less than the control counterparts. The EODF regimen resulted in lower liver levels of glycogen, glucose, and lactate, but did not affect lactate level in mouse cerebral cortex of both sexes. Activities of key glycolytic enzymes (hexokinase, phosphofructokinase, and pyruvate kinase) in liver of experimental mice were lower than those in controls. In the cerebral cortex, only hexokinase and pyruvate kinase activities were lower than in controls, but phosphofructokinase activity was not affected in IF females and was higher in IF males as compared with *ad libitum* fed males. Mitochondria isolated from liver of IF mice had lower respiratory control ratios, but those from the cortex had the same values as control animals. The concentration of β-hydroxybutyrate and the activity of β-hydroxybutyrate dehydrogenase were lower in the IF mouse liver, but not changed or enhanced in the IF cerebral cortex. Thus, animal responses to IF do not depend significantly on sex and are directed to decrease energy metabolism to save resources, and the effects are more pronounced in the liver than in the brain.

## Introduction

Under unlimited access to food, all animals including humans tend to consume excess of food that is poorly controlled due to a natural instinct to accumulate fuel reserves for use in times of food scarcity (La Fleur et al., 2014; Harris et al., 2018). Particularly in our modern times, this can lead to overweight and obesity that can cause health problems such as cancer, diabetes, cardiovascular, and neurodegenerative diseases and shortening of life and health spans (Rusli et al., 2017; Friedman, 2018). Restriction of food consumption along with physical activity (Myers et al., 2017) are effective natural strategies known to date to cope with negative consequences of overfeeding, whereas pharmacological approach to decrease weight is likely accompanied by side effects. Food intake reduced relative to an *ad libitum* (AL) diet is called dietary restriction (DR) and was found to extend mean and maximum life span and health span in a variety of organisms, ranging from bacteria to humans, and is also beneficial in relieving diverse age-related pathologies (Mattson et al., 2017; Xie et al., 2017). Such restriction has been found to decrease body mass and normalize blood glucose, insulin, and leptin levels in obese animals and humans helping at metabolic diseases including metabolic syndrome. In addition to effects on peripheral tissues, DR improves the operation of the central nervous system and weakens the symptoms of age-related neurodegenerative disorders in rodent models (Mattson et al., 2017).

Two different approaches have been used to model DR in rodents (Wahl et al., 2016; Antoni et al., 2017). In the first approach, animals receive food daily but the amount of food is reduced by 30–40% compared to what could be consumed AL. In the second approach, called intermittent fasting, animals are provided with food for certain periods of time. In this mode, they are deprived of food for a full day, every other day, or 1 day in three but in between are fed AL. A third version of DR may place organisms on an intermittent fasting schedule, on feeding days they are not given food *ad libitum*, but receive lower amounts of food than the amount consumed on the AL regimen. Interestingly, although the first two approaches to DR mentioned above look different, they frequently result in similar changes in parameters such as body mass, temperature, heart rate, blood pressure, and levels of glucose and insulin (Le Couteur et al., 2016; Hao et al., 2018; Matyi et al., 2018). Diverse molecular mechanisms are supposed to be responsible for the beneficial DR effects, including down-regulation of insulin signaling, activation of metabolic processes and biogenesis of mitochondria, a decrease in steady-state levels of reactive oxygen species (ROS), and induction of cytoprotective stress responses (Gouspillou and Hepple, 2013; Pani, 2015). Intermittent fasting was also shown to promote mitohormesis, activate mitochondrial oxidative phosphorylation, and inhibit glycolysis (O’Neill and Hardie, 2013).

Whereas the mechanisms underlying malnutrition in the adult and aged population are being intensively investigated, little is known about the mechanisms underlying malnutrition in the adolescent and juvenile age. At the same time, according to the World Health Organization globally about 41 million children are overweight or obese whereas another 155 million are chronically undernourished (World Health Statistics, 2017). Therefore, there is a need for understanding (1) how the young organism reacts to food restriction and (2) whether this reaction might turn out to be beneficial for treating overweight and obesity during adolescence. Prolonged decrease in food supply may diminish energy production; therefore, we hypothesize that intermittent fasting may be not favorable for young mice, which need more energy resources for growth than adults do. Intermittent fasting may induce metabolic reorganization in young mice to ensure with energy resources the organs, whose functions cannot be slowed down. Thus, different tissues like cerebral cortex and liver may be affected differently by intermittent fasting, but sex difference at young age could be negligible. To address these questions we chose every-other-day feeding (EODF) regimen for treatment of one-month-old mice as it does not require individual housing and enables littermates to stay as a group and measured glycolytic and mitochondrial potentials of the mouse cerebral cortex and the liver in animals of both sexes.

## Materials and Methods

### Reagents

Phenylmethylsulfonyl fluoride (PMSF), KH_2_PO_4_, NaCl, ethylenediamine tetraacetic acid (EDTA), ATP, ADP, MgCl_2,_ β-hydroxybutyrate, phosphoenolpyruvate, sodium L-lactate, sodium pyruvate, imidazole, hydrazine, aldolase, triosephosphate isomerase, glyceraldehyde 3-phosphate dehydrogenase, fructose 6-phosphate, glucose 6-phosphate, glucose, G6PDH, КСl, mannitol, bovine serum albumin, glutamate, malate, rotenone, succinate, antimycin A, ascorbate, *N,N,N*′,*N*′-tetramethyl-*p*-phenylenediamine (TMPD), and NaN_3_ were purchased from Sigma-Aldrich (St. Louis, MO, USA); Tris-HCl, NADP, NADH, NAD^+^, glycine, lactate dehydrogenase were from Carl Roth (Karlsruhe, Germany); diagnostic kits for determination of glucose and triacylglycerides were from Felicit Diagnostics Ltd. (Dnipro, Ukraine). All other reagents were obtained from local suppliers (Ukraine) and were of analytical grade.

### Animals and Experimental Conditions

The mice used were mixed C57BL/6 × sv129 strain, acquired from the Yuriy Fedkovych Chernivtsi National University (Chernivtsi, Ukraine). All animals were bred in our department facilities and housed under standard laboratory conditions (12-h light/dark photoperiod, 20–24°C temperature and 50–60% humidity).

One month old mice of both sexes were randomly allocated to the control and experimental groups. Mice in the control group had *ad libitum* access to food, whereas mice in experimental groups were subjected to an EODF regimen. Animals in the experimental group had free access to food for 24 h alternating with food deprivation for the next 24 h. Food was provided to the EODF groups at 9 a.m. and withdrawn at 9 a.m. the next day. All mice received regular chow containing 21.8% protein, 4.8% fat, 69.1% carbohydrates, and 3.9% fiber (“Rezon-1,” Kyiv, Ukraine) and had free access to water at all times. Mice were separated by sex and housed in groups of 6–7 mice per cage at the beginning of the study and kept on their respective diet regimens for 1 month. Body mass and food consumption were measured once every 6 days to minimize stress effects on the animals. All animals survived until the end of experiment. After 1 month on either control or experimental protocols, food was withdrawn for ~2 h from 9 a.m. to 11–12 a.m. prior to euthanasia. Experimental mice were sampled on the morning after their last day of fasting. All experimental procedures were approved by the Animal Experimental Committee of Vasyl Stefanyk Precarpathian National University according to NIH guidelines.

### Tissue Collection and Homogenization

Mice used for enzyme or metabolite analyses were euthanized using carbon dioxide gas anesthesia. Blood samples were quickly taken from the retro-orbital sinus and then cerebral cortex and liver were rapidly dissected and frozen in liquid nitrogen (−196°C) followed by transfer to −80°C for storage. All measurements were started with frozen tissue samples.

To determine the activity of β-hydroxybutyrate dehydrogenase (HBDH) and metabolite levels, except triacylglycerides (TAG), tissues were homogenized using a Potter-Elvehjem glass homogenizer in a 1:10 ratio (mg tissue/μl of buffer) at 4°C in medium containing (final concentrations) 50 mM potassium phosphate buffer (KPi, pH 7.0), 0.5 mM ethylenediaminetetraacetic acid (EDTA), and 1 mM phenylmethylsulfonyl fluoride (PMSF). To determine TAG content, tissue samples were homogenized in phosphate buffered saline with 0.05% Triton X-100 in a 1:10 ratio. For measurement of the activities of glycolytic enzymes, tissues were homogenized in 50 mM imidazole buffer (pH 7.5) containing 0.5 mM EDTA, 1 mM PMSF, 1 mM dithiothreitol, 20 mM NaF, and 150 mM KCl, centrifuged (16,100 *g*, 15 min, 4°C) in an Eppendorf 5415 R centrifuge (Hamburg, Germany) and supernatants were collected and used for biochemical assays. Homogenates for glycogen, glucose and TAG assays were first heated at 70°C to inactivate endogenous enzymes and then centrifuged at 12,000 *g*, 10 min, 21°C.

### Determination of Biochemical Indices

Biochemical analyses were performed using a Spekol 211 spectrophotometer (Carl Zeiss Jena, Jena, Germany). Concentrations of liver and cortex glucose, glycogen, and TAG were measured by colorimetric methods using diagnostic kits from Felicit Diagnostics Ltd. (Dnipro, Ukraine) and following the manufacturer’s instructions.

To determine lactate and pyruvate levels, tissue samples were homogenized in 0.5 M HClO_4_ at a ratio 1:10 and centrifuged (13,000 *g*, 15 min, 21°C). The acid supernatants were then neutralized with 2 M KOH. Concentrations of lactate were determined by the method of Cuddihee and Fonda (1982), based on the oxidation of lactate to pyruvate by lactate dehydrogenase and following NAD^+^ reduction at 340 nm. To prevent the reverse reaction (from pyruvate to lactate), we added hydrazine that forms a complex with pyruvate. The reaction mixture contained 0.5 M glycine-hydrazine buffer (pH 9.0), 2 U LDH, 2 mM NAD^+^ and 50 μl of neutralized supernatant in a final volume of 1 ml. Lactate solutions in a range from 10 to 160 mM were used for the calibration curve building.

Concentrations of the ketone body (KB), β-hydroxybutyrate (BHB), were measured by spectrophotometric monitoring of NADH production during conversion of BHB into acetoacetate by HBDH at wavelength 340 nm (Brashear and Cook, 1983). The reaction mixture contained 100 mM Tris-HCl buffer (pH 8.0), 1.8 mM NAD^+^, 0.5 U HBDH and 60–100 μl supernatant in a final volume of 1 ml. β-Hydroxybutyrate solutions in a range from 0.1 to 10 mM were used to build the calibration curve.

Concentrations of metabolites are expressed in milligrams per gram wet mass (mg/gwm) of tissue for glucose and TAG or micromoles per gram wet mass (μmol/gwm) for lactate, pyruvate, and BHB. Soluble protein concentrations were determined with the Coomassie Brilliant blue G-250 (Bradford, 1976), using bovine serum albumin as a standard.

Hexokinase (HK) activity was measured by monitoring NADP^+^ reduction at 340 nm in a reaction mixture containing 50 mM imidazole buffer (pH 7.5), 10 mM glucose, 5 mM MgCl_2,_ 2 mМ ATP, 0.2 mМ NADP^+^, 0.5 U G6PDH, and 30 μl of supernatant in a final volume of 1.0 ml (Lushchak et al., 1998).

Phosphofructokinase (PFK) activity was measured by monitoring NADH oxidation at 340 nm in a reaction mixture containing 50 mM imidazole buffer (pH 7.5), 5 mM fructose 6-phosphate, 5 mM MgCl_2_, 5 mМ ATP, 0.16 mМ NADH, 50 mM КСl, 0.5 U aldolase, 0.5 U triose-phosphate isomerase, 2 U glyceraldehyde-3-phosphate dehydrogenase, and 30 μl of supernatant in a final volume of 1.0 ml (Lushchak et al., 1998).

Pyruvate kinase (PK) activity was measured in a coupled reaction with LDH by monitoring NADH oxidation at 340 nm. The reaction mixture contained 50 mM imidazole buffer (pH 7.5), 1 mM phosphoenolpyruvate, 5 mM MgCl_2_, 50 mM КСl, 2.5 mM ADP, 0.16 mM NADH, 2.5 U LDH, and 5 μl of supernatant in a final volume of 1.0 ml (Lushchak et al., 1998).

Lactate dehydrogenase (LDH) activity was measured by monitoring NADH oxidation at 340 nm in a reaction mixture containing 50 mM potassium phosphate buffer (pH 7.5), 0.5 mM EDTA, 0.2 mM NADH, 1 mM pyruvate, and 10 μl of supernatant in a final volume of 1.0 ml (Lushchak et al., 1998).

Glucose-6-phosphate dehydrogenase (G6PDH) was measured by monitoring NADP reduction at 340 nm in a reaction mixture containing 50 mM potassium phosphate buffer (pH 7.5), 5 mM MgCl_2_, 2 mM glucose-6-phosphate, 0.2 mM NADP, and 10–30 μl of supernatant in a final volume of 1.0 ml.

Beta-hydroxybutyrate dehydrogenase activity was measured by monitoring NADH production at the conversion of β-hydroxybutyrate to acetoacetate at wavelength 340 nm (Bergmeyer et al., 1967; Brashear and Cook, 1983). The reaction mixture contained 100 mM Tris-HCl buffer (pH 8.0), 1.8 mM NAD^+^, 25 mM β-hydroxybutyrate and 20–80 μl supernatant in a final volume of 1 ml.

The extinction coefficient for NAD(P)H of 6.22 mM^−1^ cm^−1^ was used for calculations of all enzyme activities. One unit of enzyme activity was defined as the amount of the enzyme consuming 1 μmol of substrate or generating 1 μmol of product per minute; the activities are expressed as international units (or milliunits) per milligram of soluble protein (U/mg protein).

### Isolation of Mitochondria and Polarography

Mice were sacrificed by cervical dislocation. Mitochondria were isolated either from one cortical hemisphere (~80–100 mg) or a piece of liver of the same mass. Samples were prepared on ice and homogenized in five volumes of ice-cold isolation buffer {250 mM sucrose, 40 mM KCl, 2 mM EGTA [ethylene glycol-bis(β-aminoethyl ether)-*N*,*N*,*N*′,*N*′-tetraacetic acid], 20 mM Tris-HCl, pH 7.2} (El-Khoury et al., 2016) by 8 strokes in 2 ml Dounce glass tissue grinder (Kimble Chase, Querétaro, Mexico). The homogenates were centrifuged at 1,000 *g* for 10 min at 4°C. The resulting supernatants were then centrifuged at 10,000 *g* for 10 min at 4°C to pellet mitochondria. The mitochondrial pellets were resuspended in 30 μl of the isolation buffer. About 100 μg of protein per respirometric chamber was used for polarography on cortical mitochondria, and 300 μg of protein for liver mitochondria. Oxygen consumption was measured with a Clark oxygen electrode (Strathkelvin Instruments, North Lanarkshire, Scotland) at 37°C in buffer containing 0.3 M mannitol, 5 mM KCl, 5 mM MgCl_2_, 10 mM KPi (pH 7.2), and 1 mg/ml bovine serum albumin (El-Khoury et al., 2016), with gradual addition of substrates or inhibitors for respiratory chain (RC) complexes. The following final concentrations of substrates and inhibitors were used in series: 5 mM glutamate, 5 mM malate, 5 mM ADP, 0.75 μM rotenone, 20 mM succinate, 2.5 μM antimycin A, 2 mM ascorbate, 0.5 mM *N*,*N*,*N*′,*N*′-tetramethyl-*p*-phenylenediamine (TMPD), and 80 mM sodium azide. The following titration protocol for testing activity of respiratory chain at oxidation of different substrates was used: (1) addition of isolated mitochondria preparation into polarography chamber (2) addition of a mixture of glutamate and malate into chamber to generate NADH by means of tricarboxylic acid cycle; the rate of oxygen consumption at this stage (state 2; LEAK) we used for calculation of respiratory control ratio (RCR); (3) addition of ADP to generate state 3 of respiration; the rate of oxygen consumption at this stage is used for RCR calculation and is also represented as NADH-driven oxygen consumption (RC complexes I, III, IV, and V are involved); (4) addition of rotenone to inhibit complex I of the RC; the rate value was used for correction of succinate-driven oxygen consumption; (5) addition of succinate that provided rate of succinate-driven oxygen consumption (RC complexes II, III, IV, and V are involved); (6) addition of antimycin A to inhibit RC complex III; (7) additions of ascorbate and TMPD to pass electrons to cytochrome c and get the value of TMPD-driven oxygen consumption (cytochrome *c*, complex IV and complex V are involved); (8) addition of sodium azide to inhibit RC complex IV, and evaluate non-specific oxygen consumption (e.g., due to autoxidation of TMPD or due to operation of other oxygen-consuming enzymes not associated with RC), and correct the value of TMPD-driven oxygen consumption.

### Statistical Analysis

Statistical analysis included normality testing for all data, using Lilliefors test with Dallal-Wilkinson approximation implemented in GraphPad Prism and R 3.5.1 (package “nortest”). Normal distribution of data on mitochondrial respiration parameters were additionally assessed graphically by shapes of quantile-quantile plots and density curves (Crawley, 2015). Samples that gave *p* value less than 0.05 by Lilliefors test were assumed non-normally distributed as well as data that showed skewed or disrupted probability density curves. Homogeneity of variances (homoscedasticity) was assessed by Levene’s test implemented in R 3.5.1 (package “car”). Heteroscedastic datasets were log-transformed. Two-way analysis of variance (ANOVA) followed by multiple pairwise comparisons, using Welch’s *t* test was applied to all normally distributed data. Obtained *p* values were adjusted for multiple testing by Benjamini-Hochberg procedure. All data, except body weight, were analyzed by two-way ANOVA and represented as barplots, where each bar shows mean ± standard error of the mean (S.E.M.). Values of body weight were compared using Welch’s *t* test (between control and intermittently fasted animals) and one-way ANOVA followed by Dunnett’s test (between weights of 3-day-old and elder mice). Non-normally distributed data were analyzed by Kruskal-Wallis test followed by Dunn’s multiple comparison test (R 3.5.1, package “dunn.test”) and represented as box-and-whisker plots with marked median, first and third quartiles (box floor and ceiling, respectively). The whiskers are extended to the most extreme data points which do not exceed one and a half of interquartile range; other data points are shown as outliers.

## Results

Four-week-old mice of both sexes were subjected to 1 month intermittent fasting using an EODF regimen with experimental groups receiving food AL on 1 day and fasting on the other day. Age-matched counterparts that were fed AL throughout the experiment were used as controls.

### Body Mass and Food Consumption

**Figure 1:**
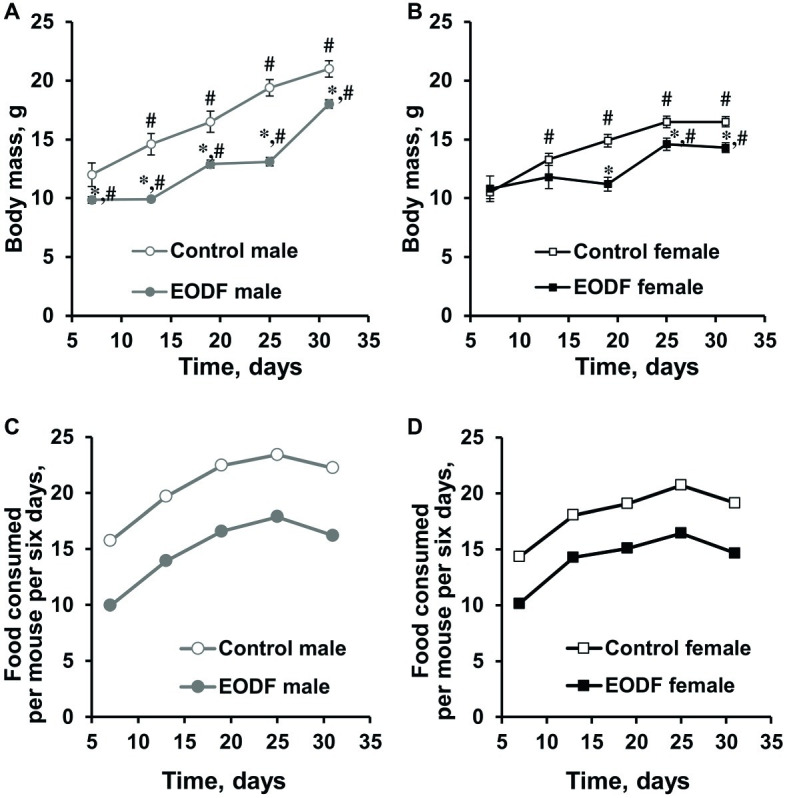
Body mass of male **(A)** and female **(B)** mice fed *ad libitum* (control, *n* = 6 males, *n* = 6 females) or subjected to an every-other-day feeding regimen (EODF, *n* = 7 males, *n* = 6 females) and the cumulative amount of food consumed per mouse for 6 days over 1 month by males **(C)** and females **(D)**. Data gathered every 6 days starting from 7th day. **(A,B)** Data are presented as mean ± S.E.M. from six to seven mice. **(C,D)** For each group, there was one cage with six to seven mice. Amount of food consumed by a group of mice in each cage was estimated each 6 days and calculated per mouse. Cumulative food consumption was calculated by summarizing data at previous and current time points. *Significantly different from the corresponding control group (*p* < 0.05), Welch’s *t* test, ^#^significantly different from initial weight of corresponding group (*p* < 0.05), Dunnett’s test after one-way ANOVA.

During the experiment, animals in all groups studied increased their body weight, but to different extents. Males of control and EODF groups virtually doubled their masses, whereas females demonstrated ~1.5-fold increase (Figures 1A,B) and this well corresponds to data from The Jackson Laboratory.^1^ The difference between masses of AL and EODF groups was observed during the experiment and EODF mice were significantly lighter than controls by 14 and 13% for males and females (*p* < 0.005, Welch’s *t* test), respectively, at the end of experiment (Figures 1A,B). At the end of experiment (31st day), control and EODF males were 27 and 26% heavier than respective females (*p* < 0.001, Welch’s *t* test).

Differences in body mass between control and EODF mice were related to the different amount of food consumed. Indeed, at the final weighing in the last week of the experiment, experimental males had consumed 73% of the food consumed by the control group (Figure 1C), whereas females had consumed 74% of their AL fed counterparts (Figure 1D). In other words, experimental animals of both sexes were dietary restricted to virtually the same extent.

### Levels of Glycolytic Substrates and Intermediates

**Figure 2:**
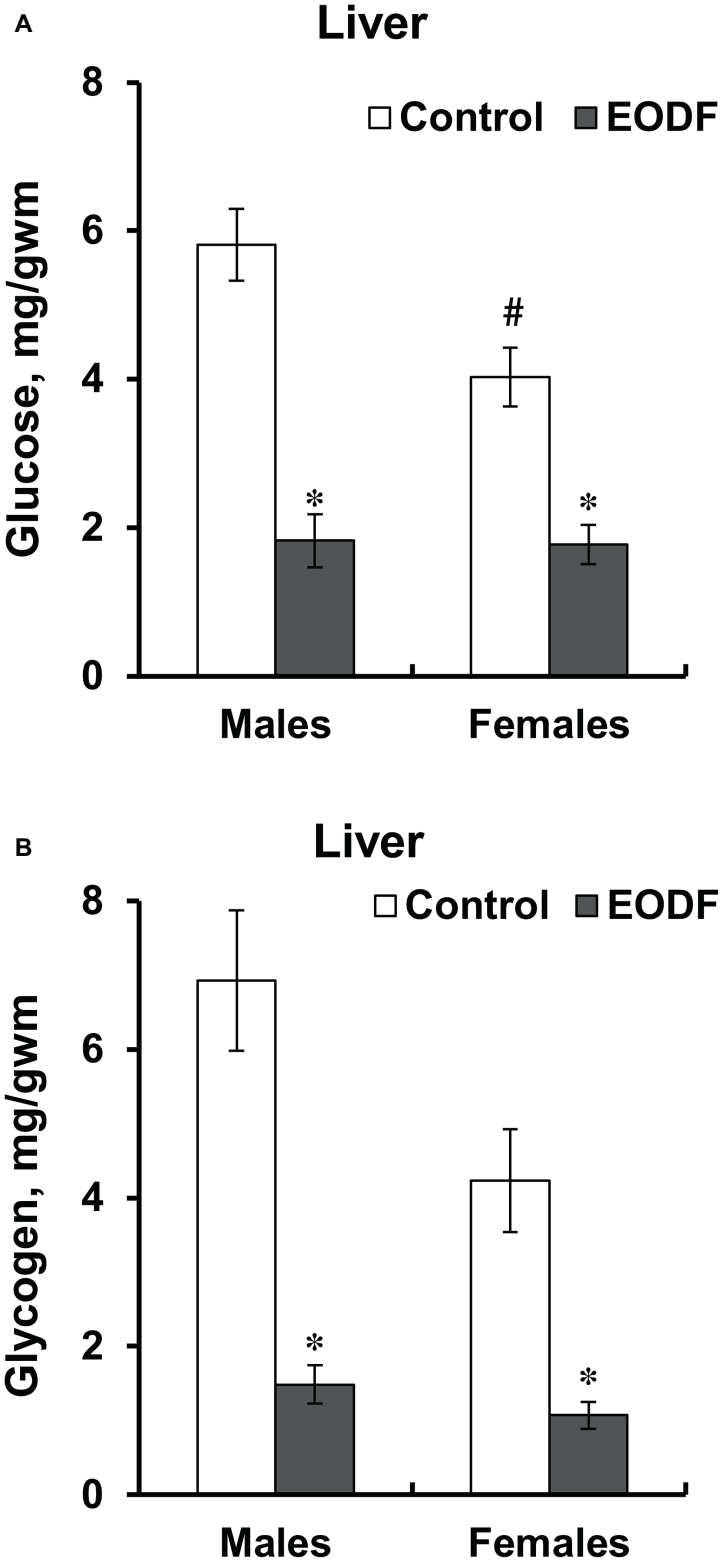
Concentrations of glucose **(A)** and glycogen **(B)** in liver of mice fed *ad libitum* (control) or subjected to an every-other-day feeding regimen (EODF) over 1 month. Here and below data are presented as mean ± SEM, *n* = 5–7 mice. *Significantly different from the control group (*p* < 0.05), ^#^significantly different from corresponding group of males (*p* < 0.05) by Welch’s *t* test with Benjamini-Hochberg adjustment of *p*.

The levels of glucose and glycogen in liver were lower in EODF mice than in controls. Liver glucose levels in EODF males and females were 32 and 44% of those in corresponding AL animals, respectively (Figure 2A). Concentrations of liver glycogen in EODF males and females were 21 and 25% of the values of AL males and females, respectively (Figure 2B). Control females had 31 and 39% lower concentrations of glucose and glycogen compared to control males, but this difference was significant only for glucose (Figures 2A,B).

**Figure 3:**
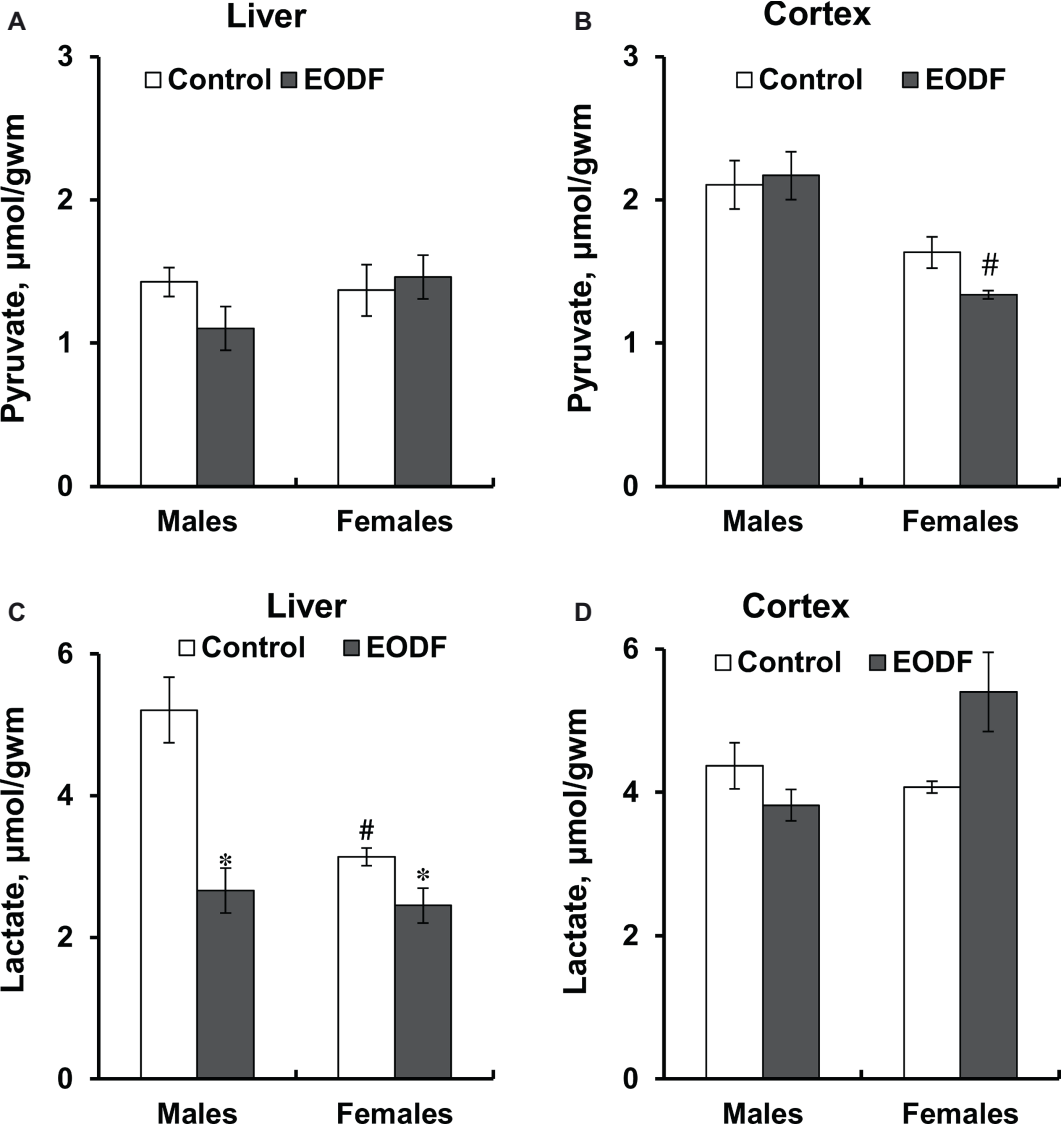
Concentrations of pyruvate **(A,B)** and lactate **(C,D)** in the liver **(A,C)** and the cortex **(B,D)** of mice fed *ad libitum* (control) or subjected to an every-other-day feeding regimen (EODF) over 1 month, *n* = 5–7 mice. *Significantly different from the control group (*p* < 0.05), ^#^significantly different from corresponding group of males (*p* < 0.05) by Welch’s *t* test with Benjamini-Hochberg adjustment of *p*.

Pyruvate levels did not differ between sexes and tissues studied as well as were affected by EODF regimen neither in males, nor in females, except in the brain cortex of EODF females they were lower by 38% than those in EODF males (Figures 3A,B). Mice subjected to EODF had significantly lower lactate concentrations in the liver than control animals: males by 50% and females by 22%, respectively, and control females had by 40% lower lactate concentrations than male counterparts (Figure 3C). In the cortex, EODF influenced lactate levels neither in males, nor in females and no sex difference in this parameter was found (Figure 3D).

In the control groups, the ratio lactate/pyruvate was highest in the liver of males and lower in the female liver, whereas in the cortex this parameter was virtually the same in males and females (Table 1). In the liver, EODF promoted a reduction in the lactate/pyruvate ratio to 71% in males and in females to 75%, but the difference was significant only for males, as compared with corresponding controls (Table 1). Females of the AL and EODF groups had by 41 and 37% lower liver lactate/pyruvate ratio than corresponding males (Table 1). In the cortex, no difference in the lactate/pyruvate ratio was found between the sexes under control conditions, but the ratio in EODF females was 54% higher than that in female controls. Lactate/pyruvate ratio in the cortex of EODF males was by 56% lower than that in EODF females.

**Table 1:** Lactate/pyruvate ratio in liver and brain.

Parameter	Tissue	Males		Females	
		Control	EODF	Control	EODF
Lactate/pyruvate ratio	Liver	3.53 ± 0.29	2.49 ± 0.26^*^	2.09 ± 0.16^#^	1.56 ± 0.16^#^
	Cortex	2.17 ± 0.26	1.72 ± 0.10	2.54 ± 0.18	3.91 ± 0.43^*^^,^^#^

*Data are presented as mean ± SEM, *n* = 5–6 mice*.

* *Significantly different from the control group*;

# *Significantly different from the corresponding values in males (*p* < 0.05)*.

### Activities of Key Glycolytic Enzymes

**Figure 4:**
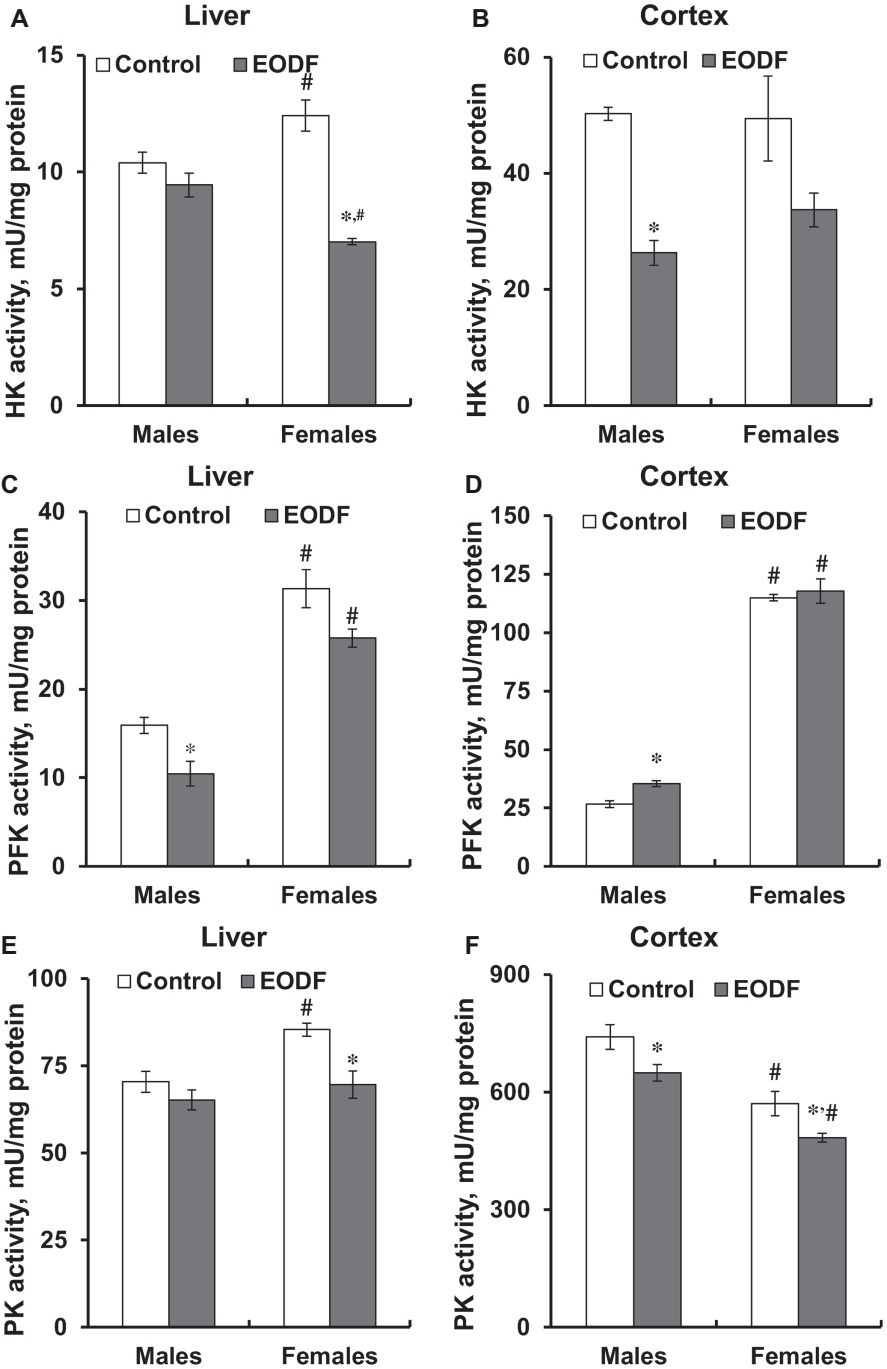
Activities of hexokinase (HK) in the liver **(A)** and the cortex **(B)**, phosphofructokinase (PFK) in the liver **(C)** and the cortex **(D)**, and pyruvate kinase (PK) in the liver **(E)** and the cortex **(F)** of mice fed *ad libitum* (control) or subjected to an every-other-day feeding regimen (EODF) over 1 month, *n* = 5–6 mice. *Significantly different from the control group (*p* < 0.05), ^#^significantly different from corresponding group of males (*p* < 0.05) by Welch’s *t* test with Benjamini-Hochberg adjustment of *p*.

First of all, it should be noted that the activities of key glycolytic enzymes, namely HK, PFK, and PK were substantially higher in the cerebral cortex compared to liver (Figure 4). In the liver of males, HK and PK activities were not influenced by EODF, whereas the activity of PFK in the EODF group was by 35% lower than that in controls. In the liver of EODF females, the activities of HK and PK were significantly lower by 43 and 19%, respectively, as compared to control mice, but PFK activity was not affected (Figures 4A,C,E). In cerebral cortex, HK activity was lower by 48% in EODF males and showed tendency to be lower in EODF females as compared to control counterparts (Figure 4B). The activity of PFK in the cortex of EODF males exceeded control values by 33%, whereas in females it was virtually the same in both control and experimental groups (Figure 4D). Finally, PK activity in the EODF mouse cortex was lower by 12 and 15% in males and females, respectively, as compared with AL mice (Figure 4E). There were differences between sexes in the activities of the key glycolytic enzymes. Particularly, HK activity was by 17% higher in the liver of AL females as compared with AL males. An opposite trend was observed for EODF females having by 25% lower HK activity than respective males. The activity of PFK in liver of AL and EODF females was by 2.0 and 2.5 fold higher than that of corresponding males. The activity of PFK was by 4.3- and 3.3-fold higher in brain cortex of AL and EODF females as compared with respective males. The activity of PK in the liver of AL females was by 18% higher compared to that of AL males. However, PK activity was by 23 and 25% lower in the cortex of AL and EODF females than that in corresponding males.

### Activities of Lactate Dehydrogenase and Glucose-6-Phosphate Dehydrogenase

Lactate dehydrogenase and G6PDH play key roles in maintaining of cellular redox ratios of NAD^+^/NADH and NADP^+^/NADPH, respectively. In our study, EODF did not significantly affect the activities of LDH and G6PDH as compared with AL mice (Table 2). However, LDH activities in liver of AL and EODF males exceeded those of females by 41 and 68%, respectively (Table 2).

**Table 2:** The activities of lactate dehydrogenase (LDH) and glucose-6-phosphate dehydrogenase (G6PDH) in mouse liver and cerebral cortex.

Parameters	Tissue	Males		Females	
		Control	EODF	Control	EODF
LDH activity (mU/mg protein)	Liver	348 ± 34	398 ± 28	247 ± 10^#^	237 ± 22^#^
	Cortex	392 ± 49	445 ± 69	521 ± 53	512 ± 42
G6PDH activity (mU/mg protein)	Liver	3.90 ± 0.60	5.59 ± 0.52	4.98 ± 0.66	4.45 ± 0.32
	Cortex	14.8 ± 1.9	12.3 ± 1.0	18.2 ± 1.93	22.8 ± 3.8

*Data are presented as mean ± SEM, *n* = 4–6 mice*.

# *Significantly different from the corresponding values in males (*p* < 0.05)*.

### Levels of Triacylglycerides, Ketone Bodies and Activity of β-Hydroxybutyrate Dehydrogenase

Concentrations of TAG in the liver demonstrated tendencies to be increased in EODF mice of both sexes, whereas in the cortex, EODF did not affect TAG levels in either sex (Table 3). In the control groups of both sexes there was no difference in TAG concentration between the liver and the cortex.

**Table 3:** Concentrations of triacylglycerides in mouse liver and brain cortex of control and EODF mice.

Parameter	Tissue	Males		Females	
		Control	EODF	Control	EODF
TAG (mg/gwm)	Liver	1.32 ± 0.11	1.58 ± 0.08	1.22 ± 0.06	1.45 ± 0.11
	Cortex	1.02 ± 0.18	0.94 ± 0.13	0.83 ± 0.06	0.95 ± 0.07

*Data are presented per gram of wet mass (gwm), mean ± SEM, *n* = 5–7 mice*.

**Figure 5:**
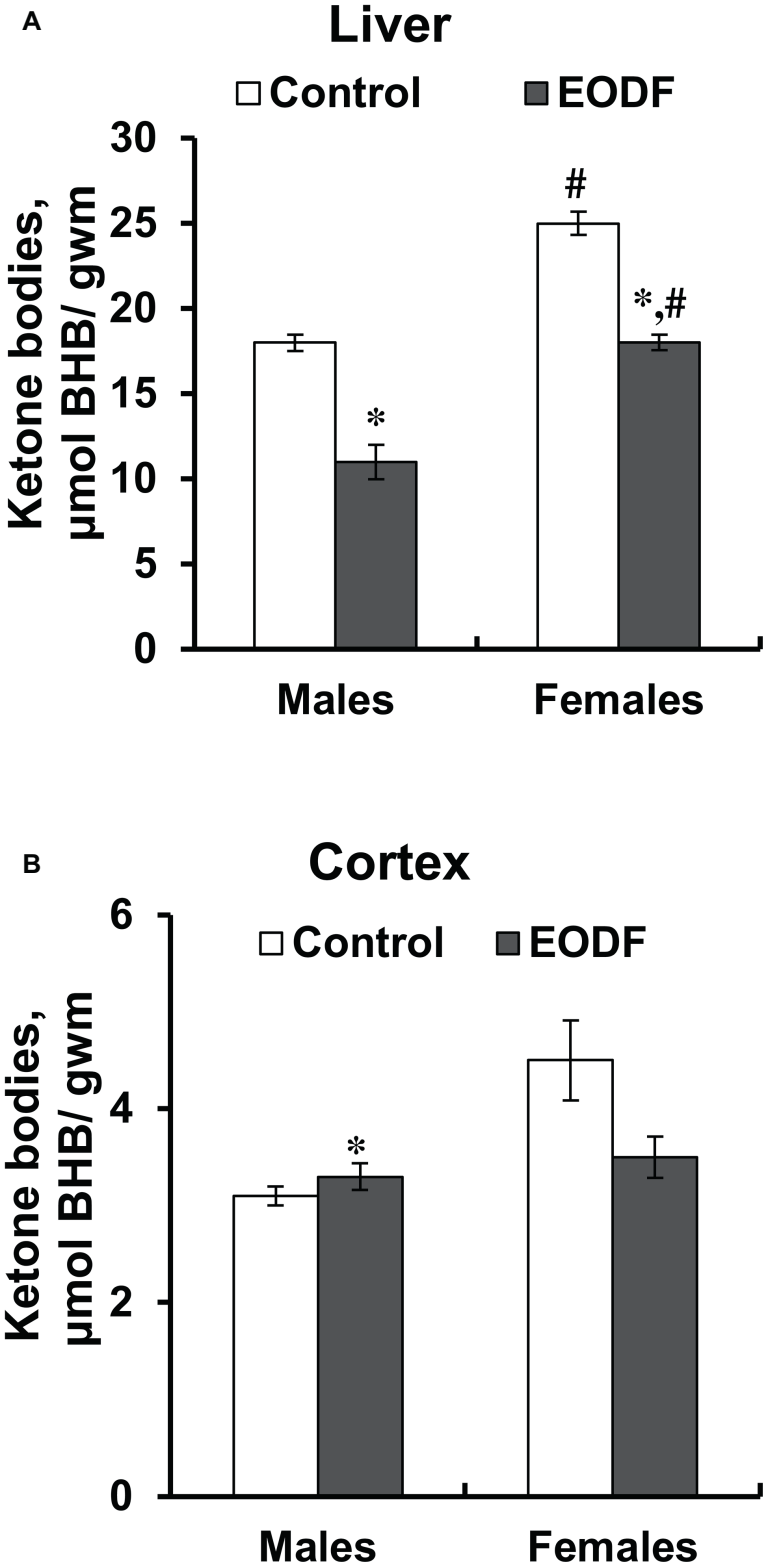
Concentrations of β-hydroxybutyrate (BHB) in liver the **(A)** and the cortex **(B)** of mice fed *ad libitum* (control) or subjected to an every-other-day feeding regimen (EODF) over 1 month, *n* = 5–6 mice. *Significantly different from the control group (*p* < 0.05), ^#^significantly different from corresponding group of males (*p* < 0.05) by Welch’s *t* test with Benjamini-Hochberg adjustment of *p*.

Concentrations of one of the ketone bodies, BHB, in the liver were substantially lower in EODF mice: by 36% in males and by 28% in females (Figure 5A). Control and EODF females had by 42 and 63% more BHB than the respective males. In the cortex, BHB concentrations in EODF males were by 43% higher compared to AL fed counterparts, but no difference was found in females (Figure 5B). Interestingly, concentrations of BHB in the liver of control mice exceeded those in the cortex by 5.6- and 7.5-fold, for males and females, respectively (Figures 5A,B).

**Figure 6:**
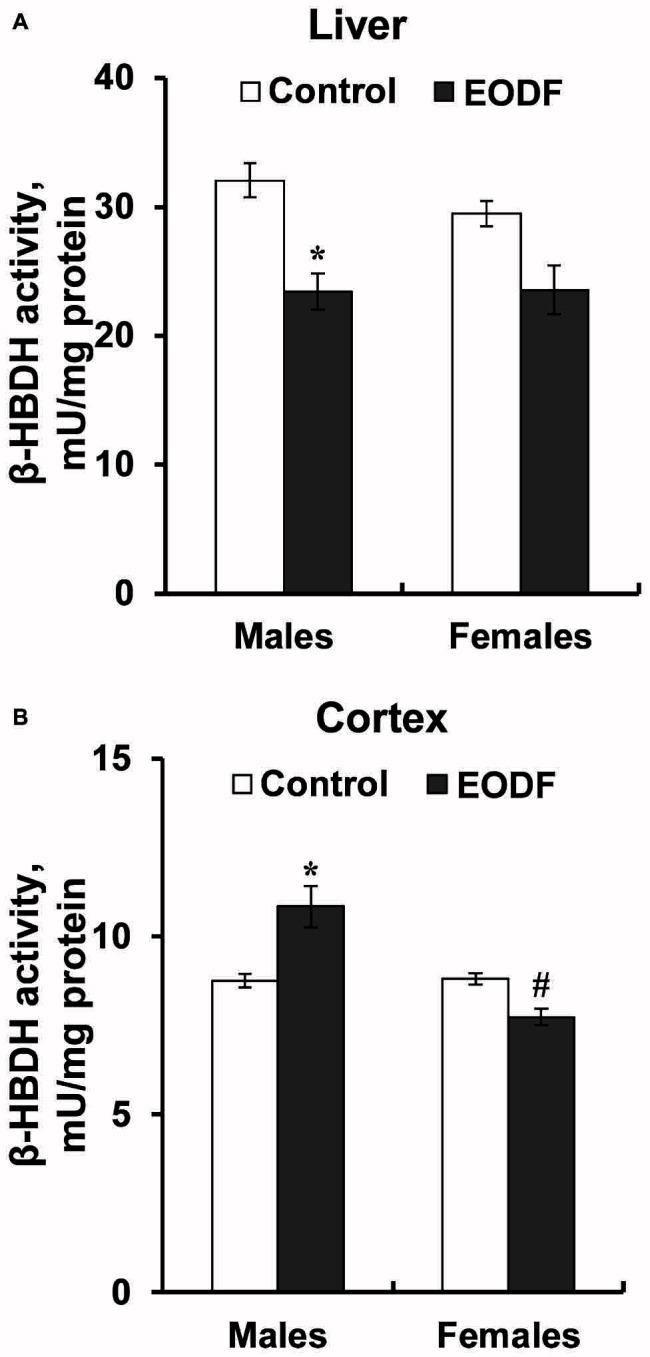
The activity of β-hydroxybutyrate dehydrogenase (HBDH) in the liver **(A)** and the cortex **(B)** of mice fed *ad libitum* (control) or subjected to an every-other-day feeding regimen (EODF) over 1 month, *n* = 5–6 mice. *Significantly different from the control group (*p* < 0.05), ^#^significantly different from corresponding group of males (*p* < 0.05) by Welch’s *t* test with Benjamini-Hochberg adjustment of *p*.

Intermittent fasting decreased the activity of HBDH in the liver of males by about 27% and did not affect it in females (Figure 6A). The activity of HBDH in the cortex of EODF males was by 23% higher than in controls, whereas this activity in EODF females was not influenced (Figure 6B). Also, EODF females had by 29% lower HBDH activity in cerebral cortex as compared with respective males.

### Operation of Mitochondria

**Figure 7:**
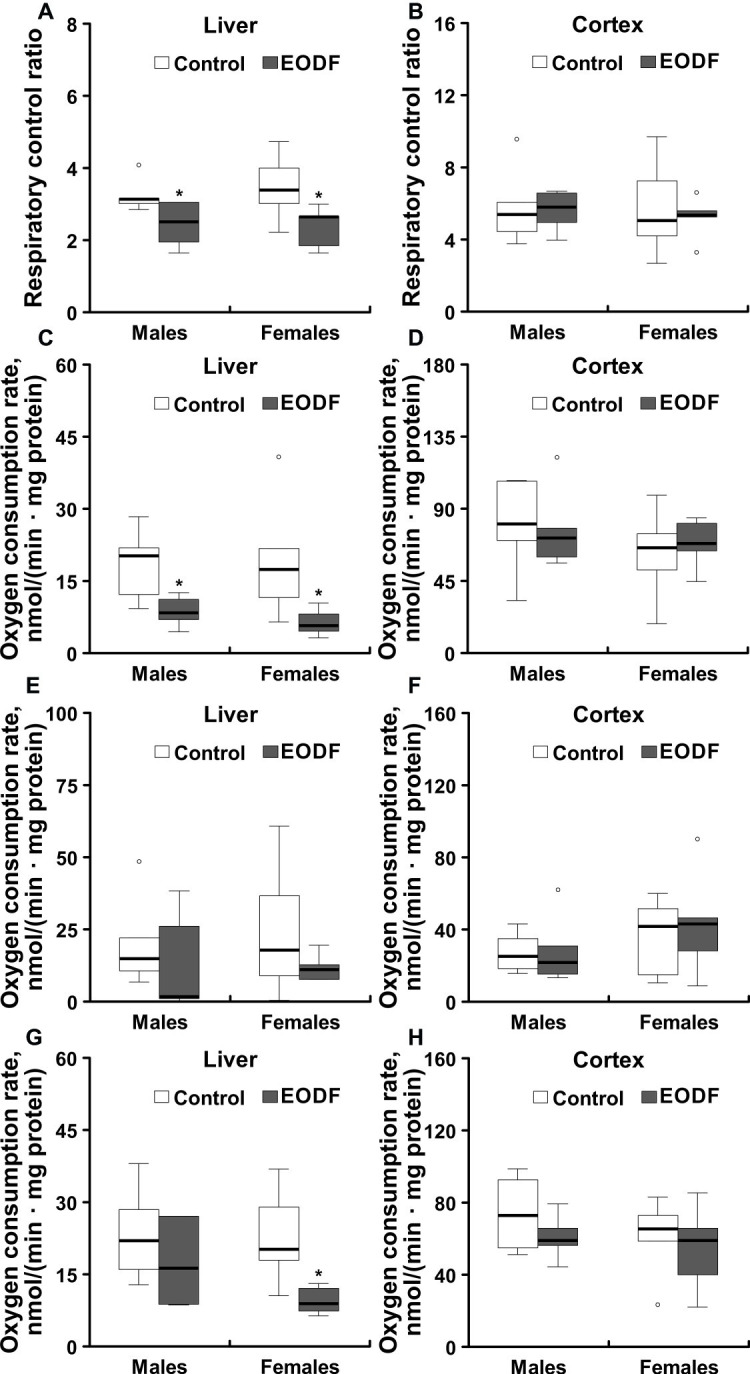
Operational parameters of mitochondria isolated from liver and cerebral cortex of *ad libitum* fed and an every-other-day feeding (EODF) mice. Respiratory control ratio of mitochondria isolated from the liver **(A)** and the cerebral cortex **(B)** of *ad libitum* fed and intermittently fasted mice; NADH-driven oxygen consumption (involving RC complexes I, III, IV, and V) of mitochondria from liver **(C)** and cerebral cortex **(D)**; succinate-driven oxygen consumption (involving RC complexes II, III, IV, and V) of mitochondria from liver **(E)** and cerebral cortex **(F)**; TMPD-driven oxygen consumption (involving cytochrome *c* and complexes IV and V) of mitochondria from liver **(G)** and cerebral cortex **(H)**.*Significantly different from the control group (*p* < 0.05) by Dunn’s test (*n* = 5–6 mice). Data are presented as median, 1st and 3rd quartiles (floor and ceiling of the boxes) ± 1.5 × interquartile range.

Liver mitochondria from males and females subjected to EODF showed RCR values that were by 22 and 31% lower as compared to the respective controls (Figure 7A). By contrast, EODF did not affect RCR of mitochondria from cerebral cortex (Figure 7B).

NADH-driven oxygen consumption (provided due to operation of RC complexes I, III, IV, and V) with glutamate and malate as substrates was significantly lower in mitochondria isolated from liver of EODF animals of both sexes compared to mitochondria of control mice (Figure 7C). In particular, NADH-driven oxygen consumption of liver mitochondria isolated from EODF animals was decreased to 47 and 34% of the values for control males and females, respectively. The same trend was seen for succinate (RC complexes II, III, IV, and V are involved) and TMPD-driven oxygen consumption (involving cytochrome *c*, RC complexes IV, and V) in males and for succinate-driven oxygen consumption in females, although the differences were not significant (Figure 7E). However, TMPD-driven oxygen consumption of mitochondria isolated from liver of EODF females was significantly lower (43% of the control value; Figure 7G). There were no substantial differences in oxygen consumption between males and females of both control and EODF groups. In addition, there were no differences between control and EODF mice in the consumption of oxygen by mitochondria isolated from cerebral cortex (Figures 7D,F,H).

## Discussion

This work differs from previous studies in two major aspects: (1) the use of one-month-old mice, this age roughly corresponds to 8–9 years of human age (Flurkey et al., 2007; Dutta and Sengupta, 2016) and (2) analyses of animals of both sexes.

### Body Mass and Food Consumption

The age of animals at the end of our experiment (8 weeks) corresponded to approximately 16–18 years of human life span (Flurkey et al., 2007; Dutta and Sengupta, 2016). Therefore, the animals were still growing, increasing their body weight in both control and experimental groups. Since many processes in young organisms differ from ones in adults, it was interesting to investigate if EODF regimen had some specific effects in such young animals. Because *ad libitum* consumption of food must directly affect energy homeostasis, we asked whether EODF influenced the central energy-providing pathways of glycolysis and oxidative phosphorylation. Since brain and liver are among main organs responsible for regulation and coordination of organism’s energetic status, the present study was focused on them.

A seminal paper which described the effects of EODF on >200 phenotype parameters in >20 mouse tissues in young and aged animals was published recently by a large international team (Xie et al., 2017). Some of the parameters evaluated by this team were also of interest to us and provided data that either supports or complements the data in the present study. In the study of Xie et al. (2017) only males of C57BL/6 mouse strain were used and a slightly different from our experimental design was followed (i.e. two-month-old mice were subjected to either 1 or 19 months of EODF). The results showed that young EODF mice tended to weigh less with lower organ masses for brain and heart, and a trend (not statistically significant) of lighter liver mass than the control groups was found (Xie et al., 2017). EODF animals were also more active, consumed more oxygen and water, and produced more carbon dioxide and heat than controls. Fasted mice also showed a lower leucocyte count, but higher numbers of red blood cells, hemoglobin, and hematocrit in blood. However, no differences in the levels of glucose, cholesterol, triacylglycerides, and activities of alkaline phosphatase (ALP) and LDH in plasma were found (Xie et al., 2017). In our previous work (Sorochynska et al., 2019), where we used the same experimental design as in the current study, blood parameters were similar to those described in the above cited work (Xie et al., 2017). In particular, we found lower leucocyte counts, circulating glucose and lactate levels, no difference in the activity of ALP, or in hemoglobin levels or erythrocyte count, whereas LDH activity was higher in males and lower in females of EODF group than that in AL groups (Sorochynska et al., 2019). The difference between these two studies may be related to factors including: (1) mouse strain specificity, (2) different animal ages, or (3) experimental design. However, most of the differences between AL fed and EODF mice were generally similar between these two studies. With number of data showing similarity between this our work and that of Xie et al. (2017), one may suggest that rest parameters which were not measured in our work could be similar to those cited in the literature. Therefore, we decided to investigate in details not only level of certain metabolites, but focused on activities of key glycolytic enzymes and mitochondria in cerebral cortex and liver in order to understand biochemical mechanisms underlying metabolic shifts at EODF.

### Tissue-Specific Response of Metabolic Fuels and Intermediates

The only work analyzing the effect of EODF on some aspects of energy providing systems was carried out in rats (Chausse et al., 2015). To obtain comparable data in mice, we have analyzed levels of main energetic substrates and metabolites, activities of key glycolytic enzymes, and the operation of mitochondria from cerebral cortex and liver of *ad libitum* and EODF young mice. Carbohydrate metabolism is usually seen as central in energy provision, especially for the brain. However, the brain is also known to use ketone bodies that were long believed to be synthesized in the liver, although it is suggested that astrocytes may also produce KB from fatty acids and transfer them to neurons (reviewed by Garaschuk et al., 2018). There is also some experimental evidence that astrocytes of *Drosophila melanogaster* can produce ketone bodies and provide them to neurons (Schulz et al., 2015).

In the present study on adolescent mice, we found that under EODF the levels of glycolytic substrates, namely glucose and glycogen, in the liver (Figures 2A,B) were lower than in control in agreement with data obtained by Xie et al. in young adult mice (Xie et al., 2017).

Some clues as to glycolytic flux are also provided by changes in the levels of glycolytic intermediates such as pyruvate and lactate (Figure 3). Whereas the concentrations of pyruvate were not changed by EODF both in the liver and cortex of males and females, EODF clearly decreased lactate concentrations in the liver of mice of both sexes (Figure 3C). Interestingly, in the cortex lactate level was not affected by EODF in males and even demonstrated a tendency to increase in females. One may interpret these data as a “feeding role” of the liver trying to provide the brain with glycolytic substrates and ketone bodies thus enabling its stable functioning. Brain is known to rely mainly on carbohydrates to meet energy demands (Jha and Morrison, 2018; Welcome and Mastorakis, 2018). Furthermore, the involvement of lactate in regulation of signaling pathways in the brain was recently described (Brooks, 2009; Jourdain et al., 2016, 2018; Dienel, 2017; Garaschuk et al., 2018). Analysis of lactate/pyruvate ratio provides further clues to energetic cooperation between the brain and the liver (Table 1). In EODF mice of both sexes, this ratio was significantly lower in the liver. This may reflect a potential shift to provision of substrates for gluconeogenesis to enhance glucose availability for the brain. But in the cortex, the ratio was not affected in males, and in females it was even substantially increased. Therefore, the redistribution of glycolytic substrates and intermediates clearly supports a potential role of the liver in providing stable fuel and energy supply for the brain.

Additional clues toward energetic cooperation between the brain and the liver are provided by triacylglycerides and ketone bodies (Table 3 and Figure 5). The concentration of TAG was not affected by the EODF regimen neither in the cortex, nor in the liver. Under conditions of limited access to food, maintenance of TAG reserves looks a logical adaptation step because TAG may serve as a precursor used for gluconeogenesis and ketogenesis (Rui, 2014; Grabacka et al., 2016). TAGs may be included in gluconeogenesis *via* at least two related processes: glycerol part can be easily converted into glycolytic intermediates such as dihydroxyacetone phosphate, whereas fatty acids can be converted into acetyl-CoA, enter citric acid cycle at the stage of oxaloacetate, i.e. can also serve as a substrate for gluconeogenesis (Weinman et al., 1957; Glew, 2010; Kaleta et al., 2011, 2012). Importantly, ketogenesis during EODF can also be important in context of gluconeogenesis. Long chain fatty acids are used to produce KB, with BHB and acetoacetate being the main ones, with certain production of acetone. Ketone bodies are produced mainly by the liver, especially under conditions of fasting regimens (Rui, 2014; Geisler et al., 2016), and are exported as fuel mainly to the brain. Stable TAG levels in EODF mice, accompanied by an increase in liver ketogenesis, allow greater adaptability to intermittent food availability (Geisler et al., 2016). A prominent role for BHB (the most abundant ketone) was well supported in the present study by direct measurement of BHB concentrations in the liver and the cortex (Figures 5A,B). Decreased BHB levels in the liver and not changed or even higher in the brain of EODF animals may be explained by the fact that the enzyme succinyl-CoA:3-ketoacid CoA transferase, needed for ketone body utilization, is not expressed in the liver but is expressed in the brain (Lukivskaya and Buko, 1993; Fukao et al., 1997; Orii et al., 2008). Due to this reason KB produced in the liver from carbohydrates and lipids are transported to other tissues particularly to the brain. Decreased activity of β-hydroxybutyrate dehydrogenase, responsible for interconversion of BHB and acetoacetate in the liver, and enhanced or not changed levels of this enzyme in the cortex (Figure 6), may indicate importance of KB in energy provision in mice exposed to EODF.

Generally, the above data indicate that under EODF conditions tight cooperation in energy provision between the liver and the brain takes place and decreased activity of β-hydroxybutyrate dehydrogenase may support export of KB from liver with its utilization in the brain.

### Selective Suppression of Key Glycolytic Enzyme Activities by Intermittent Fasting

In most cases the activities of key glycolytic enzymes, namely HK, PFK, and PK, were mainly suppressed in the liver and the cerebral cortex of EODF mice (Figure 4). Interestingly, in the liver the activities of all these enzymes were lower in EODF mice relative to controls, and only in two cases, for HK and PK in males, these reductions were not significant. In the brain of both sexes, the activities of HK and PK behaved similarly to the responses seen in the liver – they were significantly lower in experimental compared to control animals. However, PFK activity in the cortex behaved absolutely differently: it was higher in EODF compared to control males and not changed in females (Figure 4D). These data may reflect at least two things: (1) suppression of glycolysis in the liver and (2) potential partial suppression of glycolysis in the brain. With the liver the situation is largely clear: our results suggest a suppression of glycolysis along with an increase in gluconeogenesis and ketogenesis during EODF. But the situation is more complicated with the brain, where the role of gluconeogenesis is not so well understood (Glenn et al., 2015; Yip et al., 2017). The stable or even enhanced PFK activity in the cortex of EODF animals indicated a crucial role of this enzyme in brain energy provision in dietary restriction conditions. Although the activities of two other glycolytic enzymes, namely HK and PK, were lower in EODF mice cortex, PFK is generally regarded as the rate-limiting enzyme in the regulation of glycolysis. Indeed, it is well known that net glycolytic flux is usually regulated by >95% at the PFK locus, and only <5% by HK and PK (Moreno-Sánchez et al., 2008).

Which general picture arises from analysis of the measured enzyme activities? It is possible, that the EODF mouse tends to save resources and hence downregulates the activities of glycolytic enzymes in both the liver and the brain. However, because catabolism of glucose is crucial for brain function, glycolytic flux in the brain should be affected minimally. This may be achieved by decreasing the activities of various glycolytic enzymes (e.g. HK and PK), but the activity of PFK, the crucial enzyme regulating net glycolytic flux, is maintained or even enhanced. Furthermore, brain energy metabolism can also be supported under fasting conditions by the oxidation of BHB and acetoacetate. A concerted operation of glycolysis and KB oxidation can provide efficient energy supply for brain function during intermittent fasting.

### Operation of Mitochondria

The EODF-mediated diminution in the capacities of RC complexes in the liver is in line with a decrease in other metabolic parameters, such as pyruvate and ketone bodies, substrates for mitochondrial respiration. Decreased capacities of RC complexes were observed earlier in liver mitochondria of fasting rats (Sorensen et al., 2006). However, a recent study showed the opposite – higher capacity of the RC for using different substrates (Chausse et al., 2015). These discrepancies between different studies, including current one, might be explained by several reasons. Metabolism and, in particular, RC capacity is affected not only by food quantity, but also by the quality and balance between macronutrients (Solon-Biet et al., 2014). Such factors as circadian rhythm and microbiota may also impact metabolic effects of EODF and vice versa (Patterson and Sears, 2017). Fasting may also slow down metabolism which can be accompanied by a decrease in RC capacity (Brown and Staples, 2010).

The capacities of RC complexes might also depend on the balance between mitochondrial biogenesis and mitophagy, a subtype of autophagy involved in mitochondrial quality control (Stotland and Gottlieb, 2015). Fasting would expectedly stimulate autophagy *via* activation of AMP-activated protein kinase (Guarente, 2008; Stotland and Gottlieb, 2015). Nevertheless, autophagy may confer opposite effects on mitochondria. Moderate mitophagy may improve the quality of the mitochondrial network *via* removing of damaged components. The latter could lead to increase in RC capacity (Guarente, 2008; Stotland and Gottlieb, 2015). However, extensive mitophagy may result in regulated metabolic rate depression such as in physiological states of torpor or hibernation (Storey, 2015; Salin et al., 2018).

We also see that NADH- and TMPD-driven oxygen consumptions of liver mitochondria in our study were the most susceptible to EODF as compared to succinate-driven oxygen consumption. Of note, NADH- and TMPD-driven oxygen consumptions may also depend on the levels of metabolites provided with food such as flavin (for complex I) and heme (cytochrome *c* and complexes III, and IV). Also, we see that cortical mitochondria, unlike liver mitochondria, were not responsible to EODF in young mice. This may indicate that brain may have compensatory or protective mechanisms which allow keeping respiratory capacity of neural cells on sustainable level independently on food provision.

## Conclusions and Perspectives

Environmental challenges such as limited access to food require a coordinated metabolic response at organism’s level, i.e. cooperation between different organs. In this case, the brain-liver axis is crucial for the adequate organism’s response. In the present study we found that adolescent mice responded to regimen of intermittent fasting in a similar way as seen in adults and that the responses of males and females were generally similar. First of all, a slightly lower weight of experimental animals, which resulted from the reduced amount of food consumed, indicates that during growth stage general response to EODF is similar to one found in adult and old animals. Lower activities of key glycolytic enzymes and of mitochondrial respiration might lead to lower energy production. However, this suggestion contradicts data of Xie et al. (2017) who found that mice subjected to EODF consumed more oxygen than the AL fed ones, but this idea has to be tested experimentally.

At feeding day certain portion of glucose and lipids is used to meet organisms’ demand in energy and other resources, whereas excessive food consumed is accumulated in the liver in TAG and glycogen forms. At starvation day, these resources are mobilized. Interestingly, since at starvation production of very-low-density lipoprotein (VLDL) in liver is lowered, its capability to export triacylglycerides is decreased. Due to this TAGs are converted to KB. Because liver does not express key KB catabolizing enzyme succinyl-coenzyme A (CoA):3-ketoacid CoA transferase, formed ketone bodies are exported from the liver to be used in the brain. Periodic repetition of feeding/starving cycles induces adaptive responses in mice directed to economic utilization of accumulated energy resources during starvation.

Further research should be directed toward elucidation of the long term consequences (such as on life span and health span) of early life EODF. It would be also interesting to evaluate whether the lower activities of glycolytic enzymes and mitochondrial oxygen consumption conferred by EODF would influence lifelong stress resistance or the efficiency of reproduction and offspring quality. Identification of mechanisms involved would shed light on potential targets providing deleterious vs. beneficial effects of EODF at early life stages, particularly related to metabolic syndrome and may be under starvation. That is important because in developed countries substantial amount of children are overweighted, whereas in developing countries many children may suffer undernutrition.

## Data Availability Statement

The raw data supporting the conclusions of this manuscript will be made available by the authors, without undue reservation, to any qualified researcher.

## Ethics Statement

All mouse protocols were approved by the Animal Experimental Committee of Vasyl Stefanyk Precarpathian National University (Ukraine) and were conducted in accordance with the European Communities Council Directives of 24 November 1986 (86/609/ECC). The manuscript does not contain clinical studies or patient data.

## Author Contributions

VL contributed in the design of this study. OS, OK, YV, DG, and TP contributed in experiment performance. MB helped in data re-check. DG and OS helped in statistical analysis. VL and DG helped in writing the original draft. OG and KS helped in writing reviews and editing. MB helped in writing, editing, and in preparation for submission.

### Conflict of Interest

The authors declare that the research was conducted in the absence of any commercial or financial relationships that could be construed as a potential conflict of interest.

## Footnotes

^1^https://www.jax.org/jax-mice-and-services/strain-data-sheet-pages/body-weight-chart-000664

## Abbreviations

AL: *ad libitum*
BHB: β-hydroxybutyrate
DR: Dietary restriction
EODF: Every-other-day fasting/feeding
G6PDH: Glucose-6-phosphate dehydrogenase
HBDH: β-Hydroxybutyrate dehydrogenase
HK: Hexokinase
KB: Ketone bodies
LDH: Lactate dehydrogenase
PFK: Phosphofructokinase
PK: Pyruvate kinase
RC: Respiratory chain
RCR: Respiratory control ratio
TAG: Triacylglycerides.

## References

[ref1] Antoni R. Johnston K. L. Collins A. L. Robertson M. D. (2017). Effects of intermittent fasting on glucose and lipid metabolism. Proc. Nutr. Soc. 76, 361–368. 10.1017/S0029665116002986, PMID: 28091348

[ref2] Bergmeyer H. U. Gawehn K. Klotzsch H. Krebs H. A. Williamson D. H. (1967). Purification and properties of crystalline 3-hydroxybutyrate dehydrogenase from *Rhodopseudomonas spheroides*. Biochem. J. 102, 423–431. 10.1042/bj1020423, PMID: 4291491 PMC1270263

[ref3] Bradford M. M. (1976). A rapid and sensitive method for the quantification of microgram quantities of protein utilizing the principle of protein-dye binding. Anal. Biochem. 72, 289–292. 10.1016/0003-2697(76)90527-3 942051

[ref4] Brashear A. Cook G. A. (1983). A spectrophotometric, enzymatic assay for D-3-hydroxybutyrate that is not dependent on hydrazine. Anal. Biochem. 131, 478–482. 10.1016/0003-2697(83)90201-4, PMID: 6614481

[ref5] Brooks G. A. (2009). Cell-cell and intracellular lactate shuttles. J. Physiol. 587, 5591–5600. 10.1113/jphysiol.2009.178350, PMID: 19805739 PMC2805372

[ref6] Brown J. C. Staples J. F. (2010). Mitochondrial metabolism during fasting-induced daily torpor in mice. Biochim. Biophys. Acta 1797, 476–486. 10.1016/j.bbabio.2010.01.009 20080074

[ref7] Chausse B. Vieira-Lara M. A. Sanchez A. B. Medeiros M. H. G. Kowaltowski A. J. (2015). Intermittent fasting results in tissue-specific changes in bioenergetics and redox state. PLoS One 10, 1–13. 10.1371/journal.pone.0120413 PMC4352038 25749501

[ref8] Crawley M. J. (2015). Statistics: An introduction using R. 2nd Edn. Chichester: John Wiley & Sons.

[ref9] Cuddihee R. W. Fonda M. L. (1982). Concentrations of lactate and pyruvate and temperature effects on lactate dehydrogenase activity in the tissues of the big brown bat (*Eptesicus fuscus*) during arousal from hibernation. Comp. Biochem. Physiol. 73, 1001–1009. 10.1016/0305-0491(82)90350-9 7151414

[ref10] Dienel G. A. (2017). The metabolic trinity, glucose-glycogen-lactate, links astrocytes and neurons inbrain energetics, signaling, memory, and gene expression. Neurosci. Lett. 637, 18–25. 10.1016/j.neulet.2015.02.052, PMID: 25725168

[ref11] Dutta S. Sengupta P. (2016). Men and mice: relating their ages. Life Sci. 152, 244–248. 10.1016/j.lfs.2015.10.025, PMID: 26596563

[ref12] El-Khoury R. Kaulio E. Lassila K. A. Crowther D. C. Jacobs H. T. Rustin P. (2016). Expression of the alternative oxidase mitigates beta-amyloid production and toxicity in model systems. Free Radic. Biol. Med. 96, 57–66. 10.1016/j.freeradbiomed.2016.04.006, PMID: 27094492

[ref13] Flurkey K. Currer J. M. Harrison D. E. (2007). “Mouse models in aging research” in The mouse in biomedical research. 2nd Edn. eds. Fox J. G. Davisson M. T. Quimby F. W. Barthold S. W. Newcomer C. E. Smith A. L. (Burlington: Academic Press), 637–672.

[ref14] Friedman J. E. (2018). Developmental programming of obesity and diabetes in mouse, monkey, and man in 2018: where are we headed? Diabetes 67, 2137–2151. 10.2337/dbi17-0011, PMID: 30348820 PMC6198344

[ref15] Fukao T. Song X. Q. Mitchell G. A. Yamaguchi S. Sukegawa K. Orii T.. (1997). Enzymes of ketone body utilization in human tissues: protein and messenger RNA levels of succinyl-coenzyme A (CoA):3-ketoacid CoA transferase and mitochondrial and cytosolic acetoacetyl-CoA thiolases. Pediatr. Res. 42, 498–502. 10.1203/00006450-199710000-00013, PMID: 9380443

[ref16] Garaschuk O. Semchyshyn H. M. Lushchak V. I. (2018). Healthy brain aging: interplay between reactive species, inflammation and energy supply. Ageing Res. Rev. 43, 26–45. 10.1016/j.arr.2018.02.003, PMID: 29452266

[ref17] Geisler C. E. Hepler C. Higgins M. R. Renquist B. J. (2016). Hepatic adaptations to maintain metabolic homeostasis in response to fasting and refeeding in mice. Nutr. Metab. 13:62. 10.1186/s12986-016-0122-x, PMID: 27708682 PMC5037643

[ref18] Glenn T. C. Martin N. A. McArthur D. L. Hovda D. A. Vespa P. Johnson M. L.. (2015). Endogenous nutritive support after traumatic brain injury: peripheral lactate production for glucose supply via gluconeogenesis. J. Neurotrauma 32, 811–819. 10.1089/neu.2014.3482, PMID: 25279664 PMC4530391

[ref19] Glew R. H. (2010). You can get there from here: acetone, anionic ketones and even-carbon fatty acids can provide substrates for gluconeogenesis. Niger. J. Physiol. Sci. 25, 2–4. Available at: https://www.ajol.info/index.php/njps/article/view/84570. PMID: 22314896

[ref20] Gouspillou G. Hepple R. T. (2013). Facts and controversies in our understanding of how caloric restriction impacts the mitochondrion. Exp. Gerontol. 48, 1075–1484. 10.1016/j.exger.2013.03.004, PMID: 23523973

[ref21] Grabacka M. Pierzchalska M. Dean M. Reiss K. (2016). Regulation of ketone body metabolism and the role of PPARα. Int. J. Mol. Sci. 17:E2093. 10.3390/ijms17122093, PMID: 27983603 PMC5187893

[ref22] Guarente L. A. (2008). Nexus for aging, calorie restriction, and sirtuins? Cell 132, 171–176. 10.1016/j.cell.2008.01.007, PMID: 18243090 PMC2680180

[ref23] Hao Y. Tsuruda T. Sekita-Hatakeyama Y. Sakamoto S. Kitamura K. (2018). A high-fat diet is deleterious to mice under glycolysis restriction. Appl. Physiol. Nutr. Metab. 43, 419–422. 10.1139/apnm-2017-0506, PMID: 29206484

[ref24] Harris L. Hamilton S. Azevedo L. B. Olajide J. De Brún C. Waller G.. (2018). Intermittent fasting interventions for treatment of overweight and obesity in adults: a systematic review and meta-analysis. JBI Database Syst. Rev. Implement. Rep. 16, 507–547. 10.11124/JBISRIR-2016-003248, PMID: 29419624

[ref25] Jha M. K. Morrison B. M. (2018). Glia-neuron energy metabolism in health and diseases: new insights into the role of nervous system metabolic transporters. Exper. Neurol. 309, 23–31. 10.1016/j.expneurol.2018.07.009, PMID: 30044944 PMC6156776

[ref26] Jourdain P. Allaman I. Rothenfusser K. Fiumelli H. Marquet P. Magistretti P. J. (2016). L-lactate protects neurons against excitotoxicity: implication of an ATP-mediated signaling cascade. Sci. Rep. 6:21250. 10.1038/srep21250, PMID: 26893204 PMC4759786

[ref27] Jourdain P. Rothenfusser K. Ben-Adiba C. Allaman I. Marquet P. Magistretti P. J. (2018). Dual action of L-lactate on the activity of NR2B-containing NMDA receptors: from potentiation to neuroprotection. Sci. Rep. 8:13472. 10.1038/s41598-018-31534-y, PMID: 30194439 PMC6128851

[ref28] Kaleta C. de Figueiredo L. F. Schuster S. (2012). Against the stream: relevance of gluconeogenesis from fatty acids for natives of the arctic regions. Int. J. Circumpolar Health 71, 1–2. 10.3402/ijch.v71i0.18436, PMID: 22584514 PMC3417715

[ref29] Kaleta C. de Figueiredo L. F. Werner S. Guthke R. Ristow M. Schuster S. (2011). *In silico* evidence for gluconeogenesis from fatty acids in humans. PLoS Comput. Biol. 7:e1002116. 10.1371/journal.pcbi.1002116, PMID: 21814506 PMC3140964

[ref30] La Fleur S. E. Luijendijk M. C. van der Zwaal E. M. Brans M. A. Adan R. A. (2014). The snacking rat as model of human obesity: effects of a free-choice high-fat high-sugar diet on meal patterns. Int. J. Obes. 38, 643–649. 10.1038/ijo.2013.159, PMID: 23979221

[ref31] Le Couteur D. G. Solon-Biet S. Cogger V. C. Mitchell S. J. Senior A. de Cabo R.. (2016). The impact of low-protein high-carbohydrate diets on aging and lifespan. Cell. Mol. Life Sci. 73, 1237–1252. 10.1007/s00018-015-2120-y, PMID: 26718486 PMC11108352

[ref32] Lukivskaya O. Y. Buko V. U. (1993). Utilization of ketone bodies by the rat liver, brain and heart in chronic alcohol intoxication. Alcohol 28, 431–436. 10.1093/oxfordjournals.alcalc.a045408 8104400

[ref33] Lushchak V. I. Bahnjukova T. V. Storey K. B. (1998). Effect of hypoxia on the activity and binding of glycolytic and associated enzymes in sea scorpion tissues. Braz. J. Med. Biol. Res. 31, 1059–1067. 10.1590/S0100-879X1998000800005, PMID: 9777012

[ref34] Mattson M. P. Longo V. D. Harvie M. (2017). Impact of intermittent fasting on health and disease processes. Ageing Res. Rev. 39, 46–58. 10.1016/j.arr.2016.10.005, PMID: 27810402 PMC5411330

[ref35] Matyi S. Jackson J. Garrett K. Deepa S. S. Unnikrishnan A. (2018). The effect of different levels of dietary restriction on glucose homeostasis and metabolic memory. Geroscience 40, 139–149. 10.1007/s11357-018-0011-5, PMID: 29455275 PMC5964050

[ref36] Moreno-Sánchez R. Saavedra E. Rodríguez-Enríquez S. Olín-Sandoval V. (2008). Metabolic control analysis: a tool for designing strategies to manipulate metabolic pathways. J. Biomed. Biotechnol. 2008:597913. 10.1155/2008/597913, PMID: 18629230 PMC2447884

[ref37] Myers A. Gibbons C. Finlayson G. Blundell J. (2017). Associations among sedentary and active behaviours, body fat and appetite dysregulation: investigating the myth of physical inactivity and obesity. Br. J. Sports Med. 51, 1540–1544. 10.1136/bjsports-2015-095640, PMID: 27044438

[ref38] O’Neill L. A. Hardie D. G. (2013). Metabolism of inflammation limited by AMPK and pseudo-starvation. Nature 493, 346–355. 10.1038/nature11862, PMID: 23325217

[ref39] Orii K. E. Fukao T. Song X. Q. Mitchell G. A. Kondo N. (2008). Liver-specific silencing of the human gene encoding succinyl-CoA: 3-ketoacid CoA transferase. Tohoku J. Exp. Med. 215, 227–236. 10.1620/tjem.215.227, PMID: 18648183

[ref40] Pani G. (2015). Neuroprotective effects of dietary restriction: evidence and mechanisms. Semin. Cell Dev. Biol. 40, 106–114. 10.1016/j.semcdb.2015.03.004, PMID: 25773162

[ref41] Patterson R. E. Sears D. D. (2017). Metabolic effects of intermittent fasting. Annu. Rev. Nutr. 37, 371–393. 10.1146/annurev-nutr-071816-064634, PMID: 28715993 PMC13170603

[ref42] Rui L. (2014). Energy metabolism in the liver. Compr. Physiol. 4, 177–297. 10.1002/cphy.c130024, PMID: 24692138 PMC4050641

[ref43] Rusli F. Lute C. Boekschoten M. V. van Dijk M. van Norren K. Menke A. L.. (2017). Intermittent calorie restriction largely counteracts the adverse health effects of a moderate-fat diet in aging C57BL/6J mice. Mol. Nutr. Food Res. 61:5. 10.1002/mnfr.201600677, PMID: 27995741 PMC6120141

[ref44] Salin K. Villasevil E. M. Anderson G. J. Auer S. K. Selman C. Hartley R. C.. (2018). Decreased mitochondrial metabolic requirements in fasting animals carry an oxidative cost. Funct. Ecol. 32, 2149–2157. 10.1111/1365-2435.13125, PMID: 30333678 PMC6175143

[ref45] Schulz J. G. Laranjeira A. Van Huffel L. Gärtner A. Vilain S. Bastianen J.. (2015). Glial β-oxidation regulates *Drosophila* energy metabolism. Sci. Rep. 5:7805. 10.1038/srep07805, PMID: 25588812 PMC4295106

[ref46] Solon-Biet S. M. McMahon A. C. Ballard J. W. Ruohonen K. Wu L. E. Cogger V. C.. (2014). The ratio of macronutrients, not caloric intake, dictates cardiometabolic health, aging, and longevity in *ad libitum*-fed mice. Cell Metab. 19, 418–430. 10.1016/j.cmet.2014.02.009, PMID: 24606899 PMC5087279

[ref47] Sorensen M. Sanz A. Gómez J. Pamplona R. Portero-Otín M. Gredilla R. (2006). Effects of fasting on oxidative stress in rat liver mitochondria. Free Radic. Res. 40, 339–347. 10.1080/10715760500250182 16517498

[ref48] Sorochynska O. M. Bayliak M. M. Vasylyk Y. V. Kuzniak O. V. Drohomyretska I. Z. Klonovskyi A. (2019). Intermittent fasting causes metabolic stress and leucopenia in young mice. Ukr. Biochem. J. 91, 53–64. 10.15407/ubj91.01.053

[ref49] Storey K. B. (2015). Regulation of hypometabolism: insights into epigenetic controls. J. Exp. Biol. 218, 150–159. 10.1242/jeb.106369, PMID: 25568462

[ref50] Stotland A. Gottlieb R. A. (2015). Mitochondrial quality control: easy come, easy go. Biochim. Biophys. Acta 1853, 2802–2811. 10.1016/j.bbamcr.2014.12.041 25596427 PMC4501896

[ref51] Wahl D. Cogger V. C. Solon-Biet S. M. Waern R. V. Gokarn R. Pulpitel T.. (2016). Nutritional strategies to optimise cognitive function in the aging brain. Ageing Res. Rev. 31, 80–92. 10.1016/j.arr.2016.06.006, PMID: 27355990 PMC5035589

[ref52] Weinman E. O. Strisower E. H. Chaikoff I. L. (1957). Conversion of fatty acids to carbohydrate; application of isotopes to this problem and role of the Krebs cycle as a synthetic pathway. Physiol. Rev. 37, 252–272. 10.1152/physrev.1957.37.2.252, PMID: 13441426

[ref53] Welcome M. O. Mastorakis N. E. (2018). Emerging concepts in brain glucose metabolic functions: from glucose sensing to how the sweet taste of glucose regulates its own metabolism in astrocytes and neurons. NeuroMolecular Med. 20, 3281–3300. 10.1007/s12017-018-8503-0 30022304

[ref54] World Health Statistics (2017). Available at: https://www.who.int/news-room/fact-sheets/detail/malnutrition (Accessed February 16, 2018).

[ref55] Xie K. Neff F. Markert A. Rozman J. Aguilar-Pimentel J. A. Amarie O. V.. (2017). Every-other-day feeding extends lifespan but fails to delay many symptoms of aging in mice. Nat. Commun. 8:155. 10.1038/s41467-017-00178-3, PMID: 28761067 PMC5537224

[ref56] Yip J. Geng X. Shen J. Ding Y. (2017). Cerebral gluconeogenesis and diseases. Front. Pharmacol. 7:521. 10.3389/fphar.2016.00521, PMID: 28101056 PMC5209353

