# Corrigendum: Every-Other-Day Feeding Decreases Glycolytic and Mitochondrial Energy-Producing Potentials in the Brain and Liver of Young Mice

**DOI:** 10.3389/fphys.2020.00864

**Published:** 2020-07-29

**Authors:** Oksana M. Sorochynska, Maria M. Bayliak, Dmytro V. Gospodaryov, Yulia V. Vasylyk, Oksana V. Kuzniak, Tetiana M. Pankiv, Olga Garaschuk, Kenneth B. Storey, Volodymyr I. Lushchak

**Affiliations:** ^1^Department of Biochemistry and Biotechnology, Vasyl Stefanyk Precarpathian National University, Ivano-Frankivsk, Ukraine; ^2^Department of Neurophysiology, Institute of Physiology, Eberhard Karls University of Tübingen, Tübingen, Germany; ^3^Department of Biology, Carleton University, Ottawa, ON, Canada

**Keywords:** intermittent fasting, glycolytic enzymes, electron transport chain, mitochondrial respiration, ketone bodies

In the original article, there was a mistake in Figure 6 as published. The incorrect data on the level of ketone bodies in the liver and the cortex of mice fed *ad libitum* (control) or subjected to an every-other-day feeding regimen (EODF) over 1 month were placed instead of the corresponding data on β-hydroxybutyrate dehydrogenase activity. The corrected Figure 6 and the legend appear below.

**Figure 6:**
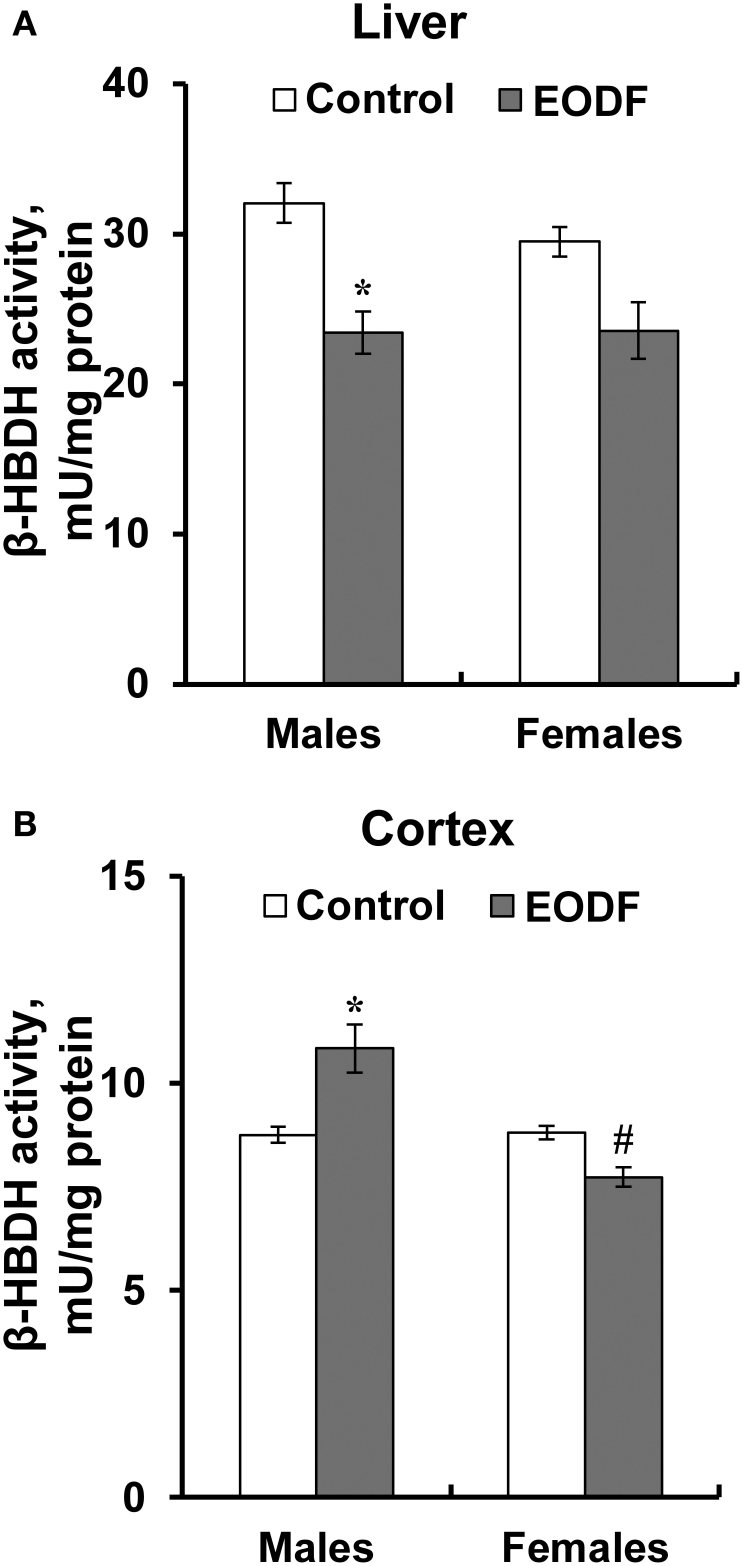
The activity of β-hydroxybutyrate dehydrogenase (HBDH) in the liver **(A)** and the cortex **(B)** of mice fed *ad libitum* (control) or subjected to an every-other-day feeding regimen (EODF) over 1 month, *n* = 5–6 mice. ^*^Significantly different from the control group (*p* < 0.05), ^#^significantly different from corresponding group of males (*p* < 0.05) by Welch's *t* test with Benjamini-Hochberg adjustment of *p*.

The authors apologize for this error and state that this does not change the scientific conclusions of the article in any way. The original article has been updated.

